# 

**Published:** 1890-11

**Authors:** 


### EDITORIAL.

### New Arrangement—Editorially.

It is very gratifying to the editor of this journal to be able to state that additional editorial workers have been secured, whereby a division of labor will be attained and the pages of the REGISTER made better and more acceptable to its readers than hitherto. This would seem to be a very natural result from the increase of work put upon it.

By reference to the first page of the cover it will be seen that two associate editors have been secured to devote some effort in this direction. These associates are Dr. Will H. Whitslar, of Youngstown, O., and Dr. N. S. Huff, of the University of Michigan, Dental Department. These gentlemen, though not as long in the field of dental literature as some others we know of, are yet very favorably known by their writing. They are both energetic, enthusiastic, and will doubtless make their mark in editorial work.

Matter for the pages of the REGISTER can be sent to either of these gentlemen, and any matters pertaining to editorial work may be addressed to them, or to the editor as heretofore.

Editorial matter from the pens of the associates will be over their respective initials. J. T.

### THE DENTAL REGISTER,

A MONTHLY MAGAZINE DEVOTED TO THE INTERESTS OF THE

**DENTAL PROFESSION.**

*Terms Reduced to* $2.00 *Per Year. Single Copies,* 25 *Cents.*

**EDITED BY PROF. J. TAFT, D.D.S.**

—AND—

**Published by SAM'L A. CROCKER & CO., Ohio Dental and Surgical Depot.**

The volume commences in January and closes in December.

The REGISTER being issued monthly is always fresh, therefore Indispensable to the practitioner who desires to keep posted up with the new inventions and improvements which are daily developed. Neither expense nor effort will be spared to give value and variety to its contents. Articles will be illustrated with well executed engravings whenever they will add to the interest or assist to explanation.

Its extensive circulation and frequency of issue make it one of the BEST DENTAL ADVERTISING MEDIUMS in the United States.

All subscriptions must begin with January or July.

Local or special notices, five lines or less, will be inserted for $3 each insertion; over five lines, 50 cts. per line.

All advertising bills payable in advance, or at furthest, after the issue of the first number containing the advertisement. All transient advertisements to be paid for in advance.

☞CONTRIBUTIONS SOLICITED.

Exclusively Editorial Communications should be addressed to the Editor.

The latest number of the REGISTER may be ordered with or without other goods at any time, and all letters should be addressed to

SAM'L A. CROCKER & CO., Ohio Dental and Surgical Depot, Cincinnati, O.

